# Effects of Lipoproteins on Metabolic Health

**DOI:** 10.3390/nu16132156

**Published:** 2024-07-06

**Authors:** Obaida Albitar, Crystal M. D’Souza, Ernest A. Adeghate

**Affiliations:** 1 Department of Pharmacology, College of Medicine and Health Sciences, United Arab Emirates University, Al Ain P.O. Box 17666, United Arab Emirates; 700040345@uaeu.ac.ae; 2 Department of Anatomy, College of Medicine and Health Sciences, United Arab Emirates University, Al Ain P.O. Box 17666, United Arab Emirates; crystal.dz@uaeu.ac.ae

**Keywords:** lipoproteins, lipid metabolism, health, homeostasis, recombinant DNA technology, diabetes mellitus, cardiovascular diseases, pharmacotherapy, inflammation, dyslipidemia

## Abstract

Lipids are primarily transported in the bloodstream by lipoproteins, which are macromolecules of lipids and conjugated proteins also known as apolipoproteins. The processes of lipoprotein assembly, secretion, transportation, modification, and clearance are crucial components of maintaining a healthy lipid metabolism. Disruption in any of these steps results in pathophysiological abnormalities such as dyslipidemia, obesity, insulin resistance, inflammation, atherosclerosis, peripheral artery disease, and cardiovascular diseases. By studying these genetic mutations, researchers can gain valuable insights into the underlying mechanisms that govern the relationship between protein structure and its physiological role. These lipoproteins, including HDL, LDL, lipoprotein(a), and VLDL, mainly serve the purpose of transporting lipids between tissues and organs. However, studies have provided evidence that apo(a) also possesses protective properties against pathogens. In the future, the field of study will be significantly influenced by the integration of recombinant DNA technology and human site-specific mutagenesis for treating hereditary disorders. Several medications are available for the treatment of dyslipoproteinemia. These include statins, fibrates, ezetimibe, niacin, PCSK9 inhibitors, evinacumab, DPP 4 inhibitors, glucagon-like peptide-1 receptor agonists GLP1RAs, GLP-1, and GIP dual receptor agonists, in addition to SGLT2 inhibitors. This current review article exhibits, for the first time, a comprehensive reflection of the available body of publications concerning the impact of lipoproteins on metabolic well-being across various pathological states.

## 1. Introduction

Various categories of lipoproteins exist, classified according to their diameter, the nature of internal cholesterol, and the existence of apolipoproteins. These include CM (chylomicrons), VLDL (very low-density lipoprotein), LDL (low-density lipoprotein), IDL (intermediate-density lipoprotein), and HDL (high-density lipoprotein). They are characterized by a unique combination of lipid species, and proteins that serve different physiological functions within the body [1]. The maintenance of cholesterol homeostasis reveals important functions in the overall biological system. The imbalances in cholesterol levels not only have implications for cardiovascular health, but also result in the development of tumors or neurological disorders. The process of esterification is a key step involving the production of cholesteryl esters from cholesterol. These esters are components of lipoproteins, thereby ensuring the stability of cholesterol levels within cells [2].

The exogenous pathway of lipoprotein formation occurs by incorporating food lipids into chylomicrons in the intestinal mucosa. As chylomicrons transport dietary triacylglycerol into the bloodstream, these chylomicrons are then broken down in the muscular system and/or adipocytes to produce chylomicron remnants that are subsequently eliminated via hepatocytes. Lipids from the diet, such as triacylglycerols, cholesteryl esters, and phospholipids, are absorbed by enterocytes lining the intestinal lumen. Enterocytes are mainly involved in the absorption of intestinal nutrients, including carbohydrates, vitamins, salts, amino acids, and lipids, through their microvilli or brush border structure.

Conversely, the synthesis of lipoproteins starts through VLDL liver production. The TG (triglycerides) present in VLDL are further processed, leading to the formation of IDL. IDL can then be subsequently converted into LDL [3]. The coexistence of diabetes mellitus and dyslipidemia greatly amplifies the risk of atherosclerotic vascular disease, leading to potential cardiovascular complications [4]. This review extensively examines the pathophysiological effects of lipoproteins, covering a wide array of topics, including their initial detection, molecular composition, and localization within various tissues. Moreover, it underscores the results obtained from studies conducted on both humans and animals, providing insights into the actions of lipoproteins, such as HDL, LDL, and VLDL in different biological systems.

### 1.1. Discovery of Lipoproteins

Throughout a span of three centuries, the understanding of a lipid transport mechanism within the plasma of mammals underwent a gradual transformation. In the early 1900s, researchers noted the presence of lecithin in plasma globulins and observed the release of small quantities of lipids from chemically crushed plasma proteins. Subsequent investigations, utilizing ultracentrifugation methods, unveiled a spectrum of lipoproteins, enabling their precise quantification. The identification of the protein constituents, known as apopeptides, occurred between 1960 and 1970, culminating in the recognition of the LDL receptor in 1974. This breakthrough led to an understanding of fat transport. Anomalies were pinpointed as the underlying cause of dyslipidemias, which played a role in the onset of conditions like atherosclerosis, xanthomatosis, and Alzheimer’s disease [5].

Research has illustrated a correlation between nutrient intake and lipid levels within the body. The findings from these studies highlight the importance of dietary choices in managing lipid levels, particularly LDL cholesterol. By incorporating foods rich in unsaturated fats, soluble fiber, and plant sterols/stanols, individuals may be able to achieve a decline in intermediate LDL levels. Conversely, certain foods like unfiltered coffee may lead to an increase in LDL cholesterol. Additional investigation may clarify how various foods affect lipid levels and provide evidence-based dietary recommendations for maintaining optimal lipid profiles [6]. In a surprising turn of events, a comprehensive study was conducted, encompassing a total of 121 experiments that analyzed data from 21,942 patients. The focus was to compare the outcomes of fourteen specific types of food against three other standard types of foods. The findings revealed that low-carbohydrate diets were not at effective as decreasing lipids with neutrally balanced foods to decrease LDL levels. However, these low-carbohydrate diets did have a positive impact on HDL cholesterol levels. Among the various named diets, namely Atkins, DASH, and Zone, it was discovered that they had the most significant effects on weight reduction and blood pressure reduction, when compared to a typical diet after a span of six months. Unfortunately, none of the diets showed significant improvements in HDL levels. These results shed light on the varying effectiveness of different diets in relation to cholesterol levels and overall health outcomes. This study serves as a valuable contribution to the field of nutrition and provides important insights into significant outcomes and limitations through various meal types. It also highlights the importance of considering not only the macronutrient composition of a diet but also its impact on specific health markers such as cholesterol levels. Further research is warranted to explore the long-term effects of these diets and to identify strategies for optimizing health outcomes through dietary interventions [7]. The findings of the study also indicated a decline in weight loss after 12 months for all types of macronutrient patterns and well-known diets, with the exception of the Mediterranean diet. Moreover, it was observed that the positive effects on heart health tended to diminish as time progressed [7]. The correlation between susceptibility to cardiovascular disease and the levels of LDL and HDL is an increasingly significant concern for global public health. To gain a wider understanding of intricate genetic traits, researchers have employed various experimental strategies. These strategies encompass classical investigations on monogenic dyslipidemias, re-sequencing techniques, and phenomic analysis. Consequently, these approaches have provided a more comprehensive model of the genetic foundation underlying plasma lipoprotein levels. This model has unveiled that multiple DNA sequence variants, both rare and common, contribute to the intricate web of genetic factors, each with varying effects. These recent advancements in genetics not only enhance our comprehension of plasma lipoprotein metabolism but also hold therapeutic aspects related to dyslipidemias [8]. Lipoproteins are subjected to endocytosis, a process where they are either broken down intracellularly, as seen in hepatocytes or macrophages [9]. In addition, some of them have been internalized and confined within the cells, possibly to carry out functions within the cell itself. Therefore, it is essential to have a comprehensive understanding of the biological roles that these lipoproteins play. Numerous studies have provided evidence suggesting that while the transportation of lipoproteins in the peripheral system has been extensively investigated, those located within the brain and spinal cord have garnered attention only in the past few years.

This increased interest is primarily due to the correlation of APOE4 with the development of Alzheimer’s disease. Consequently, there has been a surge in research focusing on LDL and neurological disorders [10]. Several investigators have indicated that atherosclerotic blood vessels are a result of LDL accumulation in the sub-endothelial layer of arteries. On the other hand, HDL works by redirecting cholesterol movement [11]. In contrast to the previously mentioned reports, alternative sources provide evidence that contradicts the information presented. These reports highlight the introduction of new therapeutic agents, such as ezetimibe, PCSK9 inhibitors, bempedoic acid, and eicosapentaenoic acid. They achieve this by targeting Lp(a), TG, and LDL. Notably, these interventions have shown promising results in improving cardiovascular outcomes [12].

The investigation conducted by Musunuru et al. has shed light on the potential of CRISPR-based editors delivered via lipid nanoparticles to make lasting changes to disease-causing genes. By utilizing this gene-editing technology, the researchers successfully modified disease-related genes in cynomolgus monkeys. The most significant outcome of this intervention was the notable reduction in PCSK9 levels in the liver. Consequently, there was a substantial decrease in both PCSK9 and LDL in the bloodstream. These positive alterations were sustained for months, indicating the promise of a “once-and-done” approach to managing LDL risk factors [13]. However, many initial observations were made about the nature and functions of lipoproteins. Current reports overwhelmingly confirm that HDL is linked to the homeostasis of body metabolism.

### 1.2. Lipoproteins Chemical Structure and Characteristics

**Figure 1:**
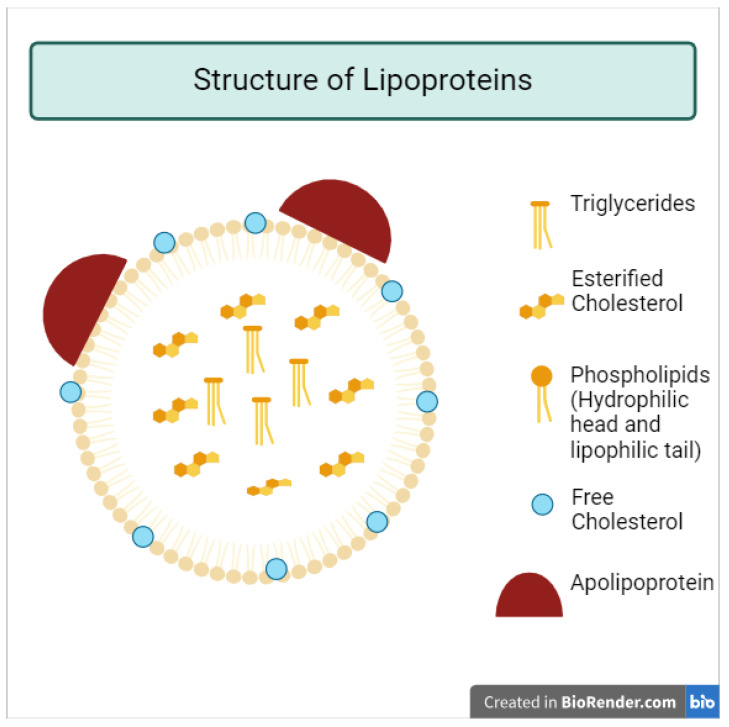
Illustrative representation of the chemical structure of lipoproteins.

Lipoproteins have a central nucleus containing triacylglycerol and cholesteryl esters. This lipid core is covered by an external polar envelope of apolipoproteins conjugated within the membrane of phospholipids and free cholesterol. This is called the amphipathic arrangement environment. It should be emphasized that lipoprotein particles engage in a constant exchange of lipids and apolipoproteins with one another, resulting in a certain level of variability in the specific composition of both lipids and apolipoproteins within each particular class. This interplay between lipids and apolipoproteins contributes to the observed heterogeneity in the actual content of lipids and apolipoproteins within lipoprotein particles (Figure 1). As mentioned, they include chylomicrons, VLDL, IDL, LDL, and HDL [14].

Figure 1 shows the chemical structure of lipoproteins. The diameters of chylomicrons range from 100 to 1200 nm, making them the largest among the lipoprotein particles. Following chylomicrons, VLDL particles have diameters ranging from 30 to 90 nm, while IDL particles have diameters, ranging from 25 to 35 nm. LDL particles have slightly smaller diameters, ranging from 18 to 25 nm, and HDL particles have the smallest diameters, measuring about 8 to 12 nm. Regarding particle density, HDL particles have the highest density among the lipoproteins, ranging between 1.063–1.21 g/mL. Nevertheless, LDL molecules have a slightly lower density, ranging within 1.019–1.063 g/mL, while IDL molecules have a density, ranging within 1.006–1.019 g/mL. VLDL molecules have a density ranging within 0.95–1.006 g/mL, whereas CM has the lowest density, measuring less than 0.95 g/mL. To summarize all of this information, while chylomicrons have the largest diameters among the lipoprotein particles, HDL particles have the smallest diameters. Conversely, HDL particles have the highest density, while chylomicrons have the lowest density among the lipoproteins.

The suggestion was made that the function of lipoproteins extends beyond lipid metabolism, prompting researchers to explore the various roles of lipoproteins. This exploration includes examining how they interact with specific elements through cancer development or its therapy [15]. It was also demonstrated that LDL may serve as a crucial link between the microvascular and macrovascular complications of diabetes, highlighting their potential significance in understanding and managing the complexities of diabetes-induced vascular diseases [16].

## 2. Functions of Lipoproteins

The gastrointestinal tract is the recipient of a variety of lipids, which originate from different sources, including the diet and bile. Lipids from the diet, such as triacylglycerols, cholesteryl esters, and phospholipids, are absorbed by enterocytes lining the intestinal lumen. Enterocytes are mainly involved in the absorption of intestinal nutrients, including carbohydrates, vitamins, salts, amino acids, and lipids, through their microvilli. These lipids are then re-esterified and packaged into pre-chylomicrons for further processing. Lipids from bile, like phospholipids and cholesterol, as well as those shed from intestinal epithelial cells, also contribute to the lipid pool in the gastrointestinal tract. The process of lipid absorption and metabolism within enterocytes involves a complex interplay of proteins and enzymes. Deficiencies in these components can result in conditions like abetalipoproteinemia and chylomicron storage disease, which are distinguished by impaired intake of lipophilic vitamins. Within the enterocyte, cytoplasmic lipid droplets serve as important storage sites for triacylglycerols, playing a key role in lipid metabolism and homeostasis. Addressing issues related to overweight may involve pharmacotherapy targeting body machinery and lipid absorption. Furthermore, emerging research highlights an intricate relationship between microbiota and lipid metabolism in the gastrointestinal tract [17].

Macronutrients involve fundamental origins to process biological ATP. Macronutrient metabolic pathways are carefully maintained within the human body. As an illustration, blood sugar has the ability to be converted into lipids by biochemical cycles. When there is an excess of lipids, they are either deposited as adipose tissues via lipoproteins. Biochemical cycles produce metabolites that are converted into various molecules within specific compartments of cells, contributing to the overall functioning of the body’s metabolic processes. Any disruptions or disorders in the metabolism of glucose and lipids can have serious consequences, potentially leading to severe health conditions like cardiac, pancreatic, or hepatic disorders. The tight range of circulating levels of glucose, fatty acids, and cholesterol underscores maintaining equilibrium for optimal functioning and overall health [18].

Each lipoprotein has a unique function; for example, chylomicron is involved in the transportation of dietary triacylglycerols [19], while VLDL is concerned with the transportation of endogenously synthesized triacylglycerols [20]. Moreover, LDL delivers cholesterol into peripheral tissues [21], whereas HDL does the opposite [22].

Furthermore, LP [a], also known as LDL, acts as an indicator of cardiovascular disease because of its ability to initiate atherosclerotic progression. There is no universally agreed-upon risk threshold; it is believed that approximately a quarter of people worldwide have lipoprotein [a] exceeding fifty milligrams per deciliter. This value is a high risk for cardiovascular complications [23], when compared to normal (<30 mg/dL). Research has shown that the progression of atherosclerotic plaque is facilitated by IDL [24]. Liu’s cohort of trials concluded that IDL acts separately from being a marker of coronary heart disease (CHD) but is a major indicator of future heart comorbidities [24].

## 3. Reverse Cholesterol Transport (RCT)

Several cardiovascular diseases in the shadow of high lipoproteins may ultimately progress to atherosclerotic coronary heart disease. A defining feature of atherosclerotic plaques is the presence of foam cells enriched with cholesterol esters. Research has suggested that HDL is a key factor in the initiation of reverse cholesterol transport (RCT). This process holds strong potential as an anti-atherogenic strategy. Despite the promising prospects of manipulating RCT for therapeutic purposes in cardiovascular disease, there was a lack of association of CVD comorbidities with HDL conventional measurement through clinical trials. Moreover, HDL and the process of RCT have shown inconsistency, adding further complexity to the matter. The challenges surrounding the utilization of RCT manipulation as a treatment approach for cardiovascular disease highlight the need for a deeper understanding of the intricate mechanisms behind heart disease. We have to understand the complex interplay between HDL particles, foam cells, and cholesterol efflux in order to develop more effective therapeutic strategies that can target atherosclerosis and its associated cardiovascular complications [25]. The excessive cholesterol produced in extra-hepatic cells can be eliminated through the cholesterol reverse transport (RCT) pathway, which is controlled by the X receptor (LXR) of the liver, along with its downstream regulators, the ATP-binding cassette subfamily A member 1 (ABCA1) and ATP-binding cassette subfamily G member 1 (ABCG1) genes. In addition, abnormal cholesterol metabolism has a significant link to the progression of diabetic retinopathy (DR). Nevertheless, the exact mechanism of the RCT pathway in the development of DR remains incompletely elucidated [26].

Apolipoprotein E (apoE) is a multifunctional protein with various roles within the body. It is involved in cholesterol efflux, particularly from cholesterol-laden macrophage foam cells and other cells relevant to atherosclerosis, as well as in reverse cholesterol transport. One of its key functions is aiding in the removal of atherogenic lipoproteins with apoB. Moreover, apoE plays a crucial role in RCT, as mentioned in the study conducted by Getz and Reardon [27]. The study showed that apoE helps to remove cholesterol from outside cells, thereby shedding light on these pathways for maintaining cardiovascular health. Furthermore, the research conducted by Getz and Reardon explores the development of apoE mimetic peptides that have the potential to enhance the processes of cholesterol efflux and reverse cholesterol transport. By investigating these peptides, researchers aim to uncover novel therapeutic strategies that could target these pathways and potentially mitigate the progression of atherosclerosis. The apoE drug analogs represent a new approach for future research on cardiovascular health and lipid metabolism [27].

The RCT pathway depends on the synchronized efforts of several proteins, including apolipoprotein A1 (ApoA1), lecithin cholesterol acyltransferase (LCAT), ATP-binding cassette transporter A1 (ABCA1), scavenger receptor-B1 (SR-B1), and free cholesterol levels [28]. The primary control of bloodstream LDL relies on macrophages, which interact with lipoproteins [29]. Lipids engulfed through phagocytosis undergo a process of transportation into digestive lysosomes. Lysosomes break down cholesteryl esters to release free cholesterol. This free cholesterol is then subjected to further modification by acetyl-CoA acetyltransferase (ACAT1), which converts it back into cholesteryl esters reserved inside the cell, particularly in the ER (endoplasmic reticulum). The cholesteryl esters stored in the endoplasmic reticulum are further converted into un-esterified cholesterol. This free cholesterol has the ability to exit the cell through cholesterol transporters. Specifically, the expression of scavenger receptors, particularly LOX-1, is increased, while the expression of cholesterol transporters is simultaneously decreased. In addition to macrophages, foam cells can also be formed from other vascular cells, which unfortunately worsens the outcome of atherosclerosis. Following the development of atherosclerosis, elevated levels of ACAT1 and reduced expression of NCEH result in the macrophage’s overload with cholesterol. This accumulation, along with the transformation of other cell types into foam cells, further exacerbates the development of atherosclerosis [29].

The study of atherosclerosis genetic progression has received substantial attention, revealing the influence of gene polymorphism and disrupted gene expression in the interplay of the immune system in processing cholesterol. This expanding body of evidence reveals genetic and molecular foundations that drive the development of this disease [30].

Another study elucidates the role of HDL receptor subtype B number 1 in facilitating the transfer of excess cholesterol to the hepatocyte plasma membrane during reverse cholesterol transport. The absence of hepatic asters proteins impedes the expulsion of cholesterol into feces, leading to a heightened bloodstream lipid limit through the accumulation of lipids in different body tissues. Hence, the aster cholesterol flux plays a crucial role in maintaining lipid balance not only in the liver but also in the entire body [31].

## 4. Lipoproteins in Health and Diseased Conditions

The correlation between LDL pathology and oxidative stress has been identified, indicating a multifaceted interaction among these elements in the progression of atherosclerosis [32]. Extensive scientific investigations have consistently demonstrated that metabolic syndrome is a risk factor for the development of CVD. Nevertheless, research specifically exploring the intricate relationship between these risk factors has uncovered a surprising opposition between Lp(a) susceptibility to acquiring diabetes mellitus [33]. Atherogenic dyslipidemia is a widespread issue observed in obese individuals, which results in the development of CVD. This form of dyslipidemia, which is linked to excessive adiposity, is characterized by low HDL with an elevation in lipoproteins that are rich in triglycerides [34]. It is apparent that low TG promotes favorable outcomes in terms of inflammation and atherosclerosis. On the other hand, high Lp(a) concentrations can elevate ASCVD, particularly in patients with very low LDL. Although statins are not used to lower Lp(a), in contrast, PCSK9 inhibitors have exhibited efficacy in reducing Lp(a) concentrations to ameliorate the diseases of the CV system [35]. Also, lipoproteins have been linked to the progression of multiple pathological disorders, including Alzheimer’s disease [36], xanthomatosis [37], breast cancer [38], fatty liver [39], peripheral neuropathy [40], pancreatitis [41], stroke [42], and deep vein thrombosis (DVT) [43].

### 4.1. Diabetes Mellitus

The established connection between insulin resistance within adipocytes in addition to alterations in TG and HDL raises questions about the independence of this relationship from liver resistance. Additionally, the impact of this association on metabolic processes like lipolysis and lipogenesis warrants continued exploration to fully comprehend the complexities of these interrelated physiological mechanisms [44]. Several studies provided further evidence that factors including heightened glycosylation, oxidative stress, and high free cholesterol levels within the circulatory system contribute to the alteration and subsequent changes in lipoprotein characteristics [45]. Numerous studies, including extensive epidemiological research, have confirmed that an excess of small, dense LDL is among the cluster of interconnected risk factors characteristic of insulin unresponsiveness [46].

A specific lipid profile is often observed in diabetic patients, known as diabetic dyslipidemia. This profile includes low HDL, high LDL, and high TG. Our primary strategy for addressing diabetic dyslipidemia revolves around lowering LDL levels, with statins being the preferred choice for treatment [47]. Furthermore, it has been shown that both ezetimibe, a cholesterol absorption inhibitor, and PCSK9 inhibitors have proven effective in reducing CV diseases and complications in diabetic patients [47].

The risk factors for microvascular complications and accelerated atherosclerosis in diabetes mellitus are multifactorial. Age and duration of diabetes play significant roles, as the longer an individual has diabetes, the higher their risk of developing complications. Genetic factors also contribute, as certain individuals may be predisposed to a higher susceptibility. Hyperglycemia, a hallmark of diabetes, induces oxidative stress, which in turn causes dyslipidemia and subsequent damage to blood vessels. Hypertension and smoking are additional risk factors that exacerbate the detrimental effects of diabetes on the vasculature of the body [48].

Statins are frequently prescribed as the primary pharmacological intervention for the management of high lipid levels. A study on a large population showed the effectiveness of statins [48]. However, it is important to note that several, alternative medications can also be utilized for this purpose. These alternative medications include ezetrol^®^, fibrates, and PCSK9 inhibitors, which offer additional options for patients with varying needs and preferences. Healthcare professionals need to stay informed about the latest developments in pharmacotherapy to provide the best possible care for patients with dyslipidemia [49]. The selection of medications depends on the individual’s lipid profile and specific lipoprotein abnormalities. Incorporating new drugs that target both hyperglycemia and lipid alterations can potentially improve diabetic dyslipidemia in these patients [49]. Lipid-lowering agents have different pharmacological actions on lipoproteins, as presented in Table 1.

### 4.2. Obesity

Obesity is characterized by the excessive storage and irregular distribution of body fat, typically accompanied by metabolic dysregulation. Dyslipidemia, a condition marked by abnormal lipid levels, is frequently associated with obesity, with apolipoproteins serving as essential structural components. Apolipoproteins have effects on developing obesity, where they exert great influence on lipid metabolism, energy utilization, and inflammatory responses [53]. The primary objective of treating dyslipidemia is to target elevated LDL concentrations [54]. During the process of evaluating CV status, it is crucial to consider the concentrations of Apo B and non-HDL lipids, which are reflective of secondary treatment aims. Weight reduction, whether through alterations in diet or the use of anti-obesity medication, results in a decline in LDL and TG levels, in addition to increasing HDL levels at the same time [54]. Additionally, the association between obesity and neuropathy has been established. Scientists have conducted studies to examine the prevalence of peripheral nerve dysfunction in individuals who are severely obese but do not have type 2 diabetes. These investigations have also explored abnormalities in lipoprotein levels [55].

The lipid profile demonstrates several remarkable changes, encompassing an upsurge in triglyceride levels, a decline in HDL concentration, and a sharp rise in LDL concentration [56]. Extensive epidemiological research has consistently demonstrated an increased occurrence of obesity in females. This finding emphasizes the critical role of obesity as a significant cause of metabolic disorders in pregnancy and, hence, the potential onset of cardiometabolic diseases later in life [56]. The comprehension of the molecular metabolism of dyslipidemia holds significant importance in reducing the negative health outcomes linked to obesity and cardiovascular diseases. Several apolipoproteins that can be interchanged, such as ApoA1, ApoA5, ApoE, and ApoC3, have been recognized for their crucial involvement in controlling lipid homeostasis and maintaining homeostatic balance in both plasma and cells. As a result, these apolipoproteins are directly involved in progressive dyslipidemia [57]. Individuals with obesity have an increased risk factor of having an MI (myocardial infarction). By acknowledging the role of obesity in cardiovascular health, efforts can be directed toward implementing preventive measures and promoting healthier lifestyles to mitigate the impact of this modifiable risk factor [58]. Bariatric surgery leads to notable enhancements in the lipid-lipoprotein profile during the early stages of the postoperative period, even before any weight loss occurs [59]. The advancements observed remain present throughout the entire follow-up period. The regression of dyslipidemia subsequent to bariatric surgery is likely facilitated by diverse mechanisms, which include positive influences on the distribution and functioning of adipose tissue, insulin responsiveness, liver health, as well as lipoprotein concentrations [59].

### 4.3. Cardiovascular Disease

**Figure 2:**
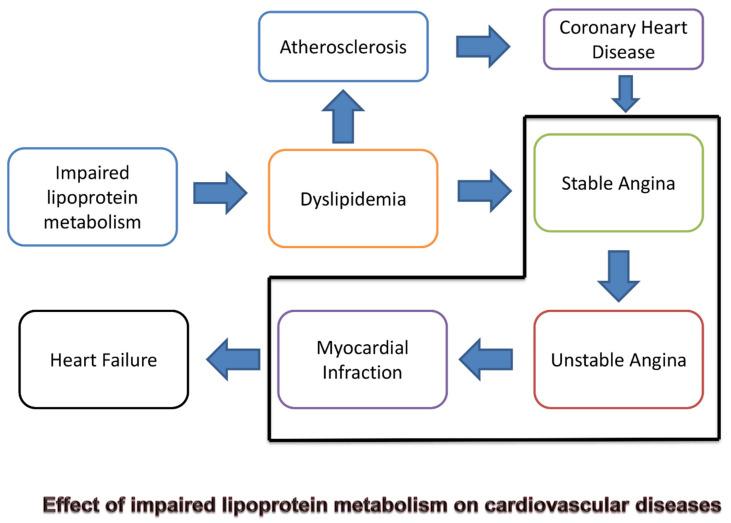
Graphical representation of the effect of impaired lipoprotein metabolism of cardiovascular diseases.

CHD is a prevalent and serious health condition that is responsible for a significant number of illnesses and deaths worldwide. The condition is characterized as a chronic disease driven by inflammation and the accumulation of lipids. Over the years, there have been remarkable advancements in understanding the intricate interactions between various risk factors associated with CHD, which led to successful protective parameters and the creation of pharmaceutical interventions that target lipoprotein-mediated risks [60]. Individuals who prioritize their cardiovascular health significantly lower their chances of developing cardiovascular disease over time. Thus, it is essential to motivate individuals to aim for optimal cardiovascular well-being, as it provides a crucial opportunity to bolster cardiovascular disease prevention efforts. Nonetheless, to prevent cardiovascular disease by endorsing optimal cardiovascular health, the strategy must pivot towards averting cardiovascular incidents by decelerating the advancement of atherosclerosis, as depicted in Figure 2. Understanding the role of atherogenic lipoproteins is essential to developing effective strategies for preventing and treating atherosclerosis [61]. The maintenance of optimal lipid levels becomes imperative for attaining ideal cardiovascular health.

While LDL cholesterol is commonly recognized and considered carefully, especially for those with CHD, it is important to keep in mind and acknowledge that other lipoproteins and their components, such as apolipoproteins, can influence the development of atherosclerosis and the incidence of cardiac events. Monitoring apo B concentrations in atherogenic lipoproteins along with apo A(I) in anti-atherogenic HDL may provide considerable value to patients’ cardiovascular health and help guide preventive strategies that lower the incidence of CVDs [62]. It is also worth noting that apolipoprotein E or apoE is linked to nearly all plasma lipoproteins and has anti-atherosclerotic properties [63].

The presence of PCSK9 in the bloodstream has been linked to the development of CVDs. This is achieved through various mechanisms, such as promoting clot formation, recruiting leukocytes, and stimulating platelet activation. These actions can contribute to the progression of cardiovascular diseases. In summary, PCSK9 modulates LDL by affecting the availability of LDL receptors. Additionally, the understanding of these functions and implications of PCSK9 can provide valuable insights into the pathophysiology of cardiovascular diseases and potential therapeutic targets for intervention [64]. Passive immunization utilizing anti-PCSK9 antibodies, including alirocumab and evolocumab, has demonstrated notable efficacy in decreasing LDL-C levels and ameliorating cardiovascular disease. This approach involves the administration of antibodies that target PCSK9 to sharply lower LDL concentrations and provide a positive impact on cardiovascular health. On the other hand, inclisiran, an RNA molecule, acts through novel interference by inhibiting the synthesis of PCSK9 [64].

### 4.4. Inflammation

Scientists and researchers have also explored the impact of lipoproteins on inflammation and their connection to diseases like atherosclerosis. Atherosclerotic lesions originate from the infiltration and alteration of lipoproteins obtained from plasma, subsequently absorbed by macrophages, to end with foam cells containing lipids, ultimately leading to the development of advanced lesions characterized by a necrotic lipid core, rendering the plaques susceptible to rupture [65]. Older age, obesity, race, or diabetes-related glucose intolerance can trigger the immune system in a setting of inflammation and oxidative stress. This process results in the development of atherosclerosis, and vasculature-related diseases [66].

The presence of oxidized LDL is a key characteristic of both hyperlipidemia and atherosclerosis. Atherosclerosis is formed by the oxidation of lipid molecules, resulting in the generation of numerous oxidized lipids. Remarkably, these same oxidized lipids are also produced within apoptotic cells. Furthermore, these abnormal lipids can be detected in many tissues and the bloodstream. Oxidized fats trigger the activation of pattern-recognition receptors, which are involved in recognizing specific molecular patterns associated with pathogens or damaged cells. Consequently, the presence of OxLDL and its associated oxidized lipids is highly related to inflammation [67]. Oxidized LDL, also known as ox-LDL, significantly worsens atherosclerosis [68]. The binding of oxidized LDL activates apoptotic pathways, increases leukocyte adhesion molecules, elevates oxidative stress, and ultimately disrupts endothelial cell function. However, in fibroblasts and vascular smooth muscle cells, ox-LDL stimulates collagen synthesis, cell proliferation, and migration [68]. Furthermore, macrophages, which play a crucial role in immune response, exhibit reduced migration capabilities and an increased tendency to form “foam cells” due to the expression of LOX-1, a protein primarily found in macrophages. LOX-1 also enhances the production of metalloproteinases, contributing to plaque instability and thrombosis.

It has been shown earlier that lipoproteins may also have a role in defending against viral, parasitic, and bacterial infections, making them a crucial part of the immune system [69]. Additionally, lipoproteins can neutralize lipopolysaccharide and lipoteichoic acid, contributing to the general detoxification processes of the body. Infections can lead to the oxidation of LDL, but oxLDL also prevents tissue damage caused by endotoxins [69]. In another study, the absence of apolipoprotein E, which is normally produced by cells, disrupts the regulation of cellular cholesterol levels, ultimately facilitating the attachment of influenza virus to cells [70]. Key serodiagnostic antigens are lipoproteins that are identified by antibodies produced during an infection. Moreover, lipoprotein vaccines have been created to stimulate an immune response aimed at managing or preventing spirochete infections [71].

## 5. Unmodifiable Risk Factors for Impaired Lipoprotein Function

### 5.1. Ageing

The aging process leads to notable alterations in lipid metabolic enzymes, which are controlled by various pathways associated with longevity. Additionally, lipids play an active role in regulating both lifespan and health span by functioning as signaling molecules. The outcome of investigations on the effect of aging on lipoprotein levels and functions is controversial. In a study involving 2128 patients, it was reported that elderly patients over the age of 75 years have a higher HDL concentration [72]. This was interpreted as being compatible with a longer life. In another large study looking at 1067 older (65–107 years) individuals in China, it was indicated that LDL-C and triglyceride levels should be monitored to prevent premature mortality [73], because they may contribute to the higher rate of atherosclerosis observed in the aged population [74].

### 5.2. Ethnicity

African ethnicity exhibits the highest levels of Lp(a) followed by South Asians, Whites, Hispanics, and East Asians, who display progressively lower levels of Lp(a) [12]. This observation may be due to genetic factors, differences in the type of diet, and other environmental factors.

### 5.3. Gender

Extensive epidemiological research has consistently demonstrated an increased occurrence of obesity in the female population. This finding emphasizes the critical role of obesity as a significant cause of metabolic disorders in pregnancy. Obesity has been associated with higher levels of total cholesterol, LDL-C, and TG. All of these factors may contribute to the potential development of cardiometabolic diseases later in life [56].

## 6. Familial Diseases of Lipoproteins

Familial plasma elevation of any of the components of lipoproteins may exist. The cause is mainly genetic. The most common form of this disease is familial hypercholesterolemia (FH). In the coming years, research will increasingly depend on site-specific mutagenesis and recombinant DNA technology to delve deeper into the similarities between function and corresponding structure, as well as the distinct functions of apolipoproteins in lipoprotein metabolism.

For example, FH arises from variations in the genetic sequences leading to an autosomal dominant inheritance pattern [75]. Individuals diagnosed with heterozygous familial hypercholesterolemia experienced notable reductions in LDL cholesterol levels when administered inclisiran, in comparison to those who were given a placebo [76].

Other diseases, like familial hypobetalipoproteinemia (FHBL), an uncommon condition, affect the metabolism of lipoproteins; individuals who are heterozygous for FHBL have lower than half the normal levels of apo B and LDL [77]. On the other hand, those who are homozygotes for FHBL have apoB levels and LDL-cholesterol that are extremely low or cannot be detected at all [77]. The identification of the genetic foundation of non-apoB FHBL may reveal promising avenues for lipid-lowering treatment [78].

The familial diseases of lipoproteins, especially the homozygous familial hypercholesterolemia (HoFH) variant, are difficult to treat. The FDA has recently approved a new drug, evinacumab, for the pharmacotherapy of HoFH [79].

## 7. Therapeutic Opportunities in Dyslipoproteinemia

In addition to traditional lipid-lowering agents, there are novel anti-diabetic medications that have shown significant promise in managing lipid levels. DPP-4 inhibitors, glucagon-like peptide-1 receptor agonists (GLP1Ras), GLP-1, and GIP dual receptor agonists, in addition to SGLT2 inhibitors, are among the newer drugs that are applied either independently or in conjunction with cholesterol-reducing drugs to achieve optimal outcomes for patients with dyslipidemia. By expanding the range of available treatment options, healthcare providers can tailor their interventions to individual patient needs and preferences. This personalized approach to managing dyslipidemia and related conditions allows for greater flexibility in treatment selection, potentially leading to improved adherence and outcomes [80,81,82,83,84,85]. Many of these drugs have varying degrees of effect. For example, in a cohort of 5963 diabetic patients who were prescribed statins, including simvastatin, they experienced a 22% decrease in vascular event rates, as indicated by a combination of the 4S study with the Air Force/Texas Coronary Atherosclerosis Prevention Study. This significant finding was derived from other extensive studies in the field [48].

## 8. Conclusions

In summary, lipoproteins, which are composed of lipids and associated proteins, act as carriers of lipids throughout the bloodstream. The processes of synthesizing lipoproteins are essential for maintaining a healthy lipid metabolism, and any disruption in these processes may result in the acquisition of various diseases. Most lipoproteins have basic structures, including a lipophilic core of triglycerides with esterified cholesterol surrounded by external phospholipids containing apolipoproteins and free un-esterified cholesterol. Lipoproteins have different densities, diameters, apolipoproteins, and lipid compositions.

Lipoproteins play an important role in exchanging dietary and endogenous lipids between tissues and organs. Recent studies suggest that every lipoprotein serves a distinct purpose; for instance, chylomicron facilitates the transportation of dietary triacylglycerols. On the other hand, VLDL is responsible for transporting endogenously synthesized triacylglycerols.

Several research studies have suggested that enhancing the elimination of cholesterol from foam cells by HDL particles holds the potential to prevent atherosclerosis. Nevertheless, the excitement surrounding the use of RCT manipulation as a treatment for CVD is diminished due to the missing correlation between CVDs and the conventional measurement of HDL cholesterol in clinical trials.

Lipoproteins are associated with different diseases, including diabetes mellitus, dyslipidemia, obesity, cardiovascular diseases, inflammation, and many others. Various pharmacological mechanisms are employed by lipid-lowering agents to target lipoproteins and achieve their desired effects. As an example, statins lower LDL, niacin increases HDL, fibrates lower triglycerides, and PCSK9 inhibitors target lipoprotein (a). Over the next few years, the field of research will rely more and more on the utilization of site-specific mutagenesis and recombinant DNA technology to treat dyslipoproteinemia.

## Figures and Tables

**Table 1:** Effects of medications on diabetes-induced dyslipidemia.

Pharmacological Class	Effect	Reference
Statins	Lower LDL levels	[47]
Fibrates	Decrease triglycerides	[50]
	Improve endothelial function	[50]
Ezetimibe	Inhibits cholesterol absorption	[47]
	Provides supportive reduction of LDL when combined with statins	[51]
Niacin	Increases HDL	[52]
PCSK9 inhibitors	Decrease lipoprotein (a)	[35]
